# Foreign Correspondence

**Published:** 1883-04

**Authors:** 


### Foreign Correspondence.

LONDON, March 10, 1883.

DEAR JOURNAL—In my last letter I gave you some account of the great hospitals of Philadelphia, and to-day I write you from "wonderful London;" truly wonderful, indeed, not only for its vastness, its magnificence and historic interest, but also for its public charities, which are commensurate with the greatness of the city. Hospitals, dispensaries, asylums, provident homes, orphanages, etc., are to be seen in every quarter of the city, and the voluntary subscriptions, donations and bequests to these institutions yearly often exceeds twenty million dollars. Most of the hospitals are free and bear conspicuously upon their walls the inscription: "Supported by Voluntary Contributions only." That there may be no lack of opportunity to give, and also, perhaps, to take advantage of the natural impulse which most of us felt when passing the outstretched hand of a beggar, most of them have fixed in closest proximity to the crowded streets a board, inscribed, "For the Support of the Hospital," around an inviting-looking aperture for coins, which are, when thus introduced, conveyed by a tin tube into the basement of the building. I am informed that a considerable revenue is derived from these "catch-pennies."

The great general hospitals, which have medical schools attached to them, are, The London, St. Bartholomew's, Guy's, Charing Cross, Middlesex, St. George's, University, King's, Westminster, St. Mary's and St. Thomas. The last named, although not the largest, far surpasses all the others in the beauty and fitness of the buildings. Indeed, there is probably no hospital in the world which, in architectural grandeur and beauty of location, can compare with it. It is approached from "the city" by the handsome Westminster bridge, the view from which is said to have suggested Wordsworth's fine sonnet, beginning: "Earth has not anything to show more fair." On one side of the river are the magnificent Parliament buildings. Opposed to them stands the hospital. It consists of eight fine buildings, in red brick, faced with stone, and united by arcades. The whole presents a river front of over 2,000 feet. These buildings were completed in 1871 at a cost of nearly two and a half millions. The existence of the hospital, however, dates back to 1215. The wards contain 600 beds and occupy the second, third and fourth stories of the buildings, the construction of which ensures complete ventilation and an abundance of light and air. Outside the main building, on the first floor, is an extensive and well-arranged out-patient department. Here the attendance of patients, including medical and surgical cases, are about 70,000 annually.

The buildings for the medical school stand at the southern extremity of the hospital, from which they are quite isolated. They contain ample accommodations for a large class of students. The dissecting rooms, and laboratories for practical physiology and chemistry are large and well appointed, and every day post mortems are made in a large hall specially constructed for the purpose.

The museum is one of the finest in the city and contains nearly all the specimens described and figured in Sir Astley Cooper's works, who was a diligent, and one of the earliest, contributors to its collection.

The pathological division alone contains over 3,000 specimens, and all are conveniently catalogued in printed volumes. Unlike most of our museums, nothing is under lock and key. The student can handle and study each specimen to his hearts content. There is also a materia medica museum, containing 600 specimens of medicinal plants, very beautifully mounted. A large reading room and library for the use of the students is constantly open and is plainly well used and enjoyed.

The faculty embraces some noted men, chief among them Bristowe, whose excellent work on "Practice" is so highly appreciated in America. I have attended some of his clinical lectures and accompanied him in his ward visits. Personally he is a man of fine presence, with a strikingly handsome and intellectual face, and courteous and gentle manners. He speaks with much fluency and without notes in rather a low but clear voice and with rapidity. Indeed, quickness is a characteristic of the man, as evidenced in every movement. In his examination of his patients he is very thorough, but gentle. His service is three times a week and extends, when I have been with him, at least from 1.30 to 5 P. M. There are a great many interesting cases in his wards. One of typhus interested me as the first I had ever seen. Bristowe states that it is here not very infrequent. At longer or shorter intervals there are outbreaks of the disease, originating mainly in the over-crowded parts of the city. Two cases of "floating kidney," both patients female and in both the right kidney implicated. He says that mobility of the kidney is much more common in women than in men, and on the right more frequently than on the left side.

I send you herewith a report of a case which may be interesting to your many readers, and in which Dr. Bristowe's diagnosis was completely confirmed by the post mortem. He predicted that a tumor would be found in the cerebellum.

W. F., aged 10; male; admitted December 12, 1882. Had been suffering for six months from headache, and vomiting three and four times a day. Complains of inability to open his eyes, and when that is over, some dimness of vision. He is a healthy-looking boy, with pain in the forehead varying in intensity. He is able to count fingers and read correctly. Pupils react to light and accommodation; no strabismus; well marked double optic neuritic. Hears, tastes and smells well; no facial paralysis. Can walk and feed himself; sensation normal; superficial reflexes normal. Heart and lungs healthy; abdomen natural. Sleeps well; no tremors.

14th. No headache; no vomiting.

16th. Still free from symptoms.

18th. Much pain in head; no vomiting. For the next ten days he continued to have more or less headache; no other symptom.

28th. Sight noticed to be defective; pain in occiput.

January 2, 1883. Sight seems to go for a few minutes and then return again.

24th. Almost completely blind, but gets up and finds his way about and seems quite sensible.

26th. Gradually increasing intervals of torpor; no sickness since admission.

February 6th. Much pain; pupils widely dilated; vomiting.

9th. Giddiness on standing first noticed.

13th. Cannot stand owing to the giddiness.

16th. Lies in a state of coma.

20th. Pain controlled by morphia. Vomits, if moved.

26th. Pain very intense; patient moaning and writhing about the bed.

27th. Much better; no pain or sickness; still giddy if he sits up.

March 4th. Had a kind of fit; head was retracted; both legs and left arm rigid; moaned, if touched; fit lasted 10 minutes; pulse almost stopped; 30 beats counted in one minute.

After this he had several more fits of the same kind, and on March 8th, at 1 A. M., 2 minims of morphia were injected subcutaneously. An hour later his breathing stopped, but the heart continued to act vigorously. Artificial respiration, with stimulant injections and enemata, kept him alive for 8½ hours longer, then the body became quite cold and the heart gradually stopped.

POST MORTEM.

Surface of convolutions, flattened; surface of brain, dry; skin, full of blood; general surface, very pale. On removing brain, the lamina terminalis and infundibulum both formed convex projections and were transparent, owing to fluid in ventricles. Both lobes of cerebellum were adherent to dura mater, and embedded in the posterior part of each lateral lobe, at about one inch from median line, was an irregular, rounded, tubercular mass, each about the size of a chestnut; the ventricles greatly distended with fluid; including, also, the third ventricle and the "iter a tertio"; brain firm and healthy in other respects; base of infundibulum formed a thick ring around the optic commissure, and the adjoining portions of optic tracts were stretched; no tubercle in membranes of brain.

The distance between the hospitals is, to a certain extent, a hindrance to good work here; but, by devoting certain days to a particular hospital, or group of hospitals, one can employ all the working hours of a day to advantage. As for material, the amount of it is something astonishing. An attendance at the service of any one of the prominent men here will occupy from two to three hours, and one may choose almost any department of medicine and find an unlimited number of cases to illustrate it.

